# Serum nucleosomes during neoadjuvant chemotherapy in patients with cervical cancer. Predictive and prognostic significance

**DOI:** 10.1186/1471-2407-5-65

**Published:** 2005-06-27

**Authors:** Catalina Trejo-Becerril, Luis F Oñate-Ocaña, Lucía Taja-Chayeb, América Vanoye-Carlo, Lucely Cetina, Alfonso Duenas-Gonzalez

**Affiliations:** 1 Unidad de Investigación Biomédica en Cáncer, Instituto Nacional de Cancerología e Instituto de Investigaciones Biomédicas (IIB/INCan), Universidad Nacional Autónoma de México (UNAM), Mexico City, Mexico; 2 Division of Surgery, Instituto Nacional de Cancerología, Mexico City, Mexico; 3 Division of Clinical Research, Instituto Nacional de Cancerología, Mexico City, Mexico

## Abstract

**Background:**

It has been shown that free DNA circulates in serum plasma of patients with cancer and that at least part is present in the form of oligo- and monucleosomes, a marker of cell death. Preliminary data has shown a good correlation between decrease of nucleosomes with response and prognosis. Here, we performed pre- and post-chemotherapy determinations of serum nucleosomes with an enzyme-linked immunosorbent assay (ELISA) method in a group of patients with cervical cancer receiving neoadjuvant chemotherapy.

**Methods:**

From December 2000 to June 2001, 41 patients with cervical cancer staged as FIGO stages IB2-IIIB received three 21-day courses of carboplatin and paclitaxel, both administered at day 1; then, patients underwent radical hysterectomy. Nucleosomes were measured the day before (baseline), at day seven of the first course and day seven of the third course of chemotherapy. Values of nucleosomes were analyzed with regard to pathologic response and to time to progression-free and overall survival.

**Results:**

All patients completed chemotherapy, were evaluable for pathologic response, and had nucleosome levels determined. At a mean follow-up of 23 months (range, 7–26 months), projected progression time and overall survival were 80.3 and 80.4%, respectively. Mean differential values of nucleosomes were lower in the third course as compared with the first course (*p *>0.001). The decrease in the third course correlated with pathologic response (*p *= 0.041). Survival analysis showed a statistically significant, better progression-free and survival time in patients who showed lower levels at the third course (*p *= 0.0243 and *p *= 0.0260, respectively). Cox regression analysis demonstrated that nucleosome increase in the third course increased risk of death to 6.86 (95% confidence interval [CI 95%], 0.84–56.0).

**Conclusion:**

Serum nucleosomes may have a predictive role for response and prognostic significance in patients with cervical cancer patients treated with neoadjuvant chemotherapy.

## Background

The search for molecular markers to predict response to treatment and prognosis in patients with cancer has always occupied a major role in cancer research. To date, glycoproteins, polypeptides, or other macromolecules such as CEA, CAI5-3, AFP, βHGC, etc. – that are determined by biochemical methods – have a definitive, albeit modest, role for patient management in some tumor types [1,2].

Knowledge of tumor biology and availability of molecular biology techniques have allowed testing of a vast array of potential molecular markers. Among these, those focused on apoptosis have gained considerable attention because of the major role that cell death plays in tumorigenesis and response to therapy [3,4].

It is well-known that free or extracellular DNA circulates in serum/plasma of patients with cancer and in healthy individuals [5]. Such an observation has been raised of late and has focused increased attention particularly on the field of tumor markers. In patients with cancer, there is often a correlation between tumor load and amount of free DNA in circulation [6]. Because part of this DNA exists in the form of oligo- and mononucleosomes, the cell death detection plus -ELISA- kit (Boehringer Mannheim) that is based on a quantitative sandwich-enzyme-immunoassay-principle using mouse monoclonal antibodies directed against DNA and histones, respectively, were developed as an *in vitro *test; nonetheless, this subsequently has been applied to quantification of oligo and mononucleosomes in tumors [7] and in the plasma/serum [8] of cancer patients. We recently demonstrated in mice and humans that at least part of nucleosomes in circulation originates from tumor cells, that the early rise of nucleosomes observed in patients undergoing chemotherapy is the consequence of the tumor apoptosis induced by chemotherapy, and that due to an efficient mechanism of depuration a lower level of nucleosomes post-chemotherapy indicates a favorable tumor response in patients with cervical cancer [9].

To date, the majority of studies that have evaluated the nucleosome level pre- and post-therapy with the enzyme-linked immunosorbent assay (ELISA) method have been performed on a small and rather heterogeneous group of patients with regard to tumor type and chemotherapy schedule [10,11]. Consequently, the potential value of serial determination of serum nucleosomes to predict response to therapy and prognosis remains to be investigated. In this study, we have addressed this issue by performing pre- and post-chemotherapy analyses of serum nucleosomes in a homogeneous group of patients with locally advanced cervical cancer treated with paclitaxel and carboplatin as neoadjuvant therapy followed by surgery.

## Methods

Forty one untreated patients with histologic diagnosis of cervical carcinoma and FIGO staged as IB2-IIIB were studied. Patients needed to meet the following inclusion criteria: 1) aged between 18 and 70 years; 2) performance status 0–2 according to Word Health Organization (WHO) criteria; 3) normal hematological, renal, and hepatic function according to standard parameters; 6) a normal chest x-ray, and 7) signature on written informed consent. Exclusion criteria included 1) severe systemic or uncontrolled disease (infection, central nervous system, metabolic, etc.) that precluded use of chemotherapy, 2) pre-existing neuropathy due to any cause, 3) pregnancy and lactation, 4) mental illness, and 5) previous or concomitant malignancies (exception, melanoma skin cancer). The protocol was approved by the regulatory boards of the Instituto Nacional de Cancerología (INCan) in Mexico City.

Treatment. Treatment consisted of carboplatin at a dose of 6 area under the curve (AUC) diluted in 500 mL of 5% glucose administered over 1 h followed by paclitaxel at 175 mg/m^2 ^administered over 3 h, both drugs on day l; a total of three 21-day courses were administered. Complete blood cell counts and biochemical profiles were performed at days 14 and 21 of each cycle. After neoadjuvant chemotherapy, patients were submitted to radical hysterectomy. Adjuvant post-operative chemoradiation with cisplatin was performed in all cases with positive surgical margins, one or more positive pelvic lymph node, and in patients with disease in parametria (all high-risk factors for recurrence) [12], as well as in cases whose residual disease contained vascular or lymphatic permeation and/or deep invasion into the middle or internal thirds of the cervical stroma (both intermediate-risk factors for recurrence) [13].

Blood sampling for nucleosome measurements. The day prior to beginning the first course of chemotherapy, a sample of 6 mL of blood was drawn from the patient's arm and centrifuged within the next h at 1,500 *g *for 20 min to obtain serum. Subsequently, we added 10 rnM EDTA (pH8) to the serum and samples were stored at -20°C until analysis. The same procedure was performed for taking and processing samples at day 7 of the first course of chemotherapy and at day 7 of the third course of chemotherapy (hereafter referred as the first and third courses).

Analysis of nucleosomes by ELISA. This assay was carried out with the cell death detection kit plus -ELISA- (Roche) as follows: 20 μL of serum samples (diluted 1:4 with incubation buffer) were placed into a streptavidin-coated microtiterplate and incubated with a mixture of anti-histone-biotin and anti-DNA-POD for 2 h at 15–25°C. Antibodies bind to the histones and DNA of nucleosomes and fix the immunocomplexes to the microtiterplates by the streptavidin-biotin interaction. After the incubation period, unbound antibodies are removed by washing with incubation buffer; then, the ABTS (2,2-Azino di-3-ethylbenzthiazolin-sulfonate) solution is added for 30 min. Incubation of the retained POD-linked complexes with ABTS as substrate permits the spectrophotometrical quantification of the nucleosomes at 405 nm. As positive control, we used the complex DNA-histone provided in the kit and as negative control, the substrate solution only. We constructed a standard curve with serial dilutions of apoptotic material ranging from 3,500 arbitrary units (AU) to 200. All determinations were performed by duplicate, and then the values of the double absorbance measurements of the samples were averaged. Afterward, background value of the immunoassay from each of these averages was subtracted and final values were expressed as AU; interassay variability was 10%.

Tumor response criteria and efficacy assessment. Response to neoadjuvant chemotherapy was evaluated pathologically in the surgical specimens and classified as complete or partial. Complete response was considered when there was no evidence of tumor cells or when there was residual microscopic disease in absence of positive pelvic or para-aortic lymph nodes, disease in parametria, positive surgical margins, vascular and/or lymphatic permeation, or deep invasion into the middle or internal thirds of the cervical stroma. Partial pathologic response was considered with any macroscopic residual, or any microscopic residual plus at least one high-risk and/or intermediate-risk factor for recurrence. Time to progression was considered from the date that chemotherapy began until the date of confirmed progressive and/or recurrent disease, whereas overall survival was considered from date of diagnosis until the date of patient's death.

Statistical analysis. Baseline level of nucleosomes was compared with those obtained at first and third courses of chemotherapy using the paired *t *test. Patients were divided into two groups depending on the differential values of nucleosomes above and below the median to correlate with pathologic response using the chi square test. Survival analysis for time to progression and overall survival was performed using the Kaplan-Meier method [14]. Differences in survival between groups were analyzed by the log-rank method [15]. Cox proportional hazards regression analysis was used to assess prognostic significance of a number of covariates in a multivariate setting [16]. Two-tailed probability values of "conventional" 0.05 or less were considered significant.

Analysis was performed with SPSS for Windows version 10 (SPSS, Inc., Chicago, IL, USA, 1999).

## Results

### Patient characteristics

A total of 41 patients were studied. Characteristics of these patients are shown in Table 1. Median patient age was 45 years (range, 24–67 years); the majority of patients had squamous tumors (8 %) and were staged IB2-IIB(84%).

### Serum nucleosomes

**Figure 1:**
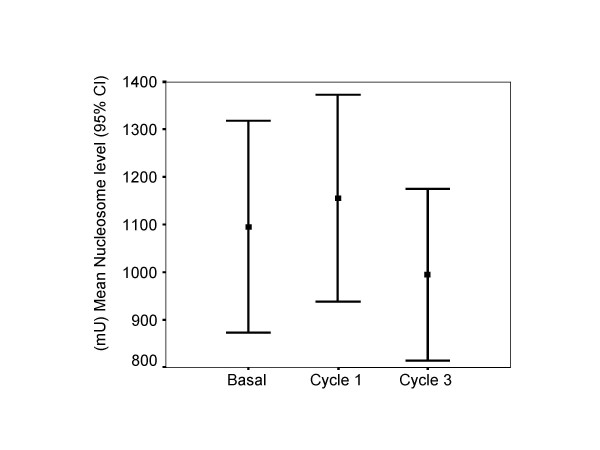
Nucleosome values. Expressed as mean values and CI 95%, had a mild increase in the first course to decrease thereafter, however, these differences were not statistically significant.

We evaluated nucleosome levels in all 41 patients before (baseline) and post-treatment at first and third chemotherapy courses. As shown in Figure 1, mean (CI 95%) baseline level was 1,085 AU (median, 825) with a range from 419–2,980 AU. Measurement at the first chemotherapy course showed a mean of 1,120 AU (CI 95%), (median, 891) ranging from 499–3,200 AU, and at the third course there was a mean of 978 AU (CI 95%) (median, 897) with a range of 471–2,930 AU. There were no statistically significant differences as compared with baseline values or between these. To further evaluate nucleosome behavior during treatment, differential values of nucleosomes were calculated between baseline and first and third courses. In the first course, levels diminished in 20 patients to a mean of -411 (standard deviation [SD] 692) and a median of -170 AU, whereas values increased in 21 patients with a mean of 561 (SD, 492) and median of 417 AU. On the other hand, during the third chemotherapy course the mean value for the 20 patients in whom values decreased was -611 (SD, 628) and the median of -327 AU, while in the remaining patient values mean increase was 430 (SD, 462) with a median of 217 AU. The paired *t *test was statistically significant (*p *<0.0001).

### Pathologic response and clinical outcome

Induction chemotherapy was well tolerated. A total of 123 cycles were administered, of which only two were delayed for 1 week. Most common toxicities were nausea/vomiting and neuropathy, which were mainly grades 1–2. There were no cases of leukopenia grade 3 but neutropenia grades 3 and 4 were present in 12 and 3% of the courses, respectively. Other toxicities were mild and uncommon. All patients completed the three chemotherapy courses and underwent radical hysterectomy. Surgical specimen analysis showed complete pathologic response in 16 cases (39%), while partial pathologic response was found in 25 of 41 patients (61%). At a mean follow-up of 23 months (range, 7–26 months), projected progression time and overall survival were 80.3 and 80.4%, respectively.

### Correlation of nucleosome levels with pathologic response

For establishing associations between pathologic response and prognosis, patients were divided into two groups depending on differential values of nucleosomes above and below the median. Nucleosome levels at first chemotherapy course showed no statistically significant correlation with response, (χ^2 ^test, *p *= 0.160) (Table 2). On the other hand, there was a statistically significant – albeit marginal – correlation between response and nucleosome levels measured at the third course, and there were 11 complete responses among the 20 patients who had lowered nucleosomes and 16 partial responses in patients with elevated nucleosomes (χ^2 ^test, *p *= 0.041) (Table 3).

### Correlation of nucleosome levels with clinical outcome

Nucleosome levels at first chemotherapy course failed to correlate with outcome. Contrariwise, survival analysis conducted with values obtained at the third course showed a statistically significant better time to progression and overall survival in patients who demonstrated lower levels (*p *= 0.0243) and *p *= 0.0260, respectively) (Figure 3). Clinical stage and tumor size were also analyzed. Earlier staged (IB2-IIB) patients had longer time to progression and survival as compared with patients staged at IIIB (*p *= 0.0164 and *p *= 0.0105, respectively), whereas patients with smaller tumors had longer time to progression and survival (*p *= 0.0096, and *p *= 0.0089, respectively). In addition, the status of complete pathologic response showed a trend for better time to progression and survival times (*p *= 0.0998 and *p *= 0.0953, respectively) (data not shown).

We assessed the prognostic significance of a number of covariates in a multivariate setting. Covariates considered for inclusion were nucleosome values at third course, stage, tumor size, and pathologic response. A forward selection procedure was used to find the combination of pairs of covariates that were significant or that showed a trend (*p *<0.1) in survival. Cox regression analysis of all patients showed that nucleosome levels and tumor size were significant prognostic factors for survival. Response rate (RR) showed that nucleosome increase in the third course increased risk of death to 6.86 (CI 95%, 0.84–56.04), whereas a tumor size >30 cm^2 ^increased risk of death to 9.08 (CI 95%, 1.11–74.20) (Table 4).

## Discussion

The majority of anticancer agents in use at present were developed using empirical screens designed to identify agents that selectively kill tumor cells. However, until recently pathologists noticed that radiation and chemotherapy can induce cell death with the morphologic features of apoptosis [17], although the significance of these observations was not widely appreciated. Currently, it is well-established that anticancer agents induce apoptosis and that disruption of apoptotic programs can reduce treatment sensitivity [18]. This connection between response to treatment and apoptosis has led to evaluation of apoptosis levels by diverse methods the levels before or during treatment with variable results. While single or baseline levels of apoptosis, or key pro- or anti-apoptotic gene products, yield mixed results in its prognostic significance [7,19-25], evaluation of the treatment-induced apoptotic effect has shown to be more useful [26,27]; however, this is associated with the risk and discomfort of repeated biopsies. In this scenario, measurement of serum nucleosomes by ELISA, a simple and non-invasive test, has facilitated the testing of its prognostic significance in cancer treatment.

Previous studies of serum nucleosomes in patients with cancer have shown that high heterogeneity in baseline levels of nucleosomes among the different types of cancer and even among patients with the same tumor type. Our present data from a homogeneous population of patients with cervical cancer confirm these observations: values ranged from 419–2,980 AU. These values are nevertheless very similar to those found by our group in a previous study in patients with cervical cancer similar clinical stages, which ranged from 627–2,313 AU [9]. On the other hand, we found no correlation of baseline levels with clinical-pathologic factors. It appears that this correlation could be tumor type-specific, as reported by Holdenrieder et al., who showed a highly significant correlation between stage and serum nucleosomes in colon but not in breast carcinoma [28].

To evaluate the potential prognostic significance of serum nucleosomes, it is important to choose adequate evaluation time points that would reflect the proapoptotic effect of chemotherapy; to date, optimal time points for measurement have not been established. Holdenrieder et al. analyzed a total of 42 patients (colorectal, lung, and lymphoma) receiving primary adjuvant of second-line chemotherapy with a variety of agents and schedules (administration in 1–5 days). Despite such heterogeneity in the study, a pattern of nucleosome increase within the first 3 days to a subsequent nucleosome decrease in the treatment-free period emerged, as well as correlation of post-treatment levels of nucleosomes with response [10]. Similar data was reported by Kuroi et al., in 18 patients with recurrent breast carcinoma undergoing treatment with docetaxel every 3–4 weeks; these authors found a statistically significant correlation between decrease in nucleosomes and response. In addition, they evaluated the kinetics of nucleosomes before and 72 h thereafter in four patients who received single-agent docetaxel; in this instance, the authors found a peak of nucleosomes at 24 h and a decrease at 72 h [11]. To the contrary, we previously reported that in patients with cervical cancer receiving oxaliplatin, gemcitabine at day 1 the rise occurred within the first hours to decrease at 24 h in the responding patients [9]. Based on these data, we decided to evaluate here nucleosomes at day 7 of the first and third courses of treatment with carboplatin and paclitaxel. At such time points, it could be expected that no significant drug levels would be in circulation and that therefore, nucleosomes would reflect tumor mass or activity as the maximum level of cytotoxicity could be attained in the days prior to measurement, as demonstrated in a murine model of tumors treated with platinum [29] or paclitaxel [30]. Interestingly, as a whole there were no nucleosome reductions either in the first course (mean baseline, 1,085 vs. 1,120) or at the third treatment course (baseline, 1,085 vs. 978). However, differential levels showed a higher decrease in the third than in the first course as compared with the baseline (-611 vs. -411, respectively). These results would suggest that as a whole, tumor reduction induced by one chemotherapy course was insufficient to produce significant reduction in nucleosome levels in this patient population receiving the carboplatin plus paclitaxel combination.

**Figure 2:**
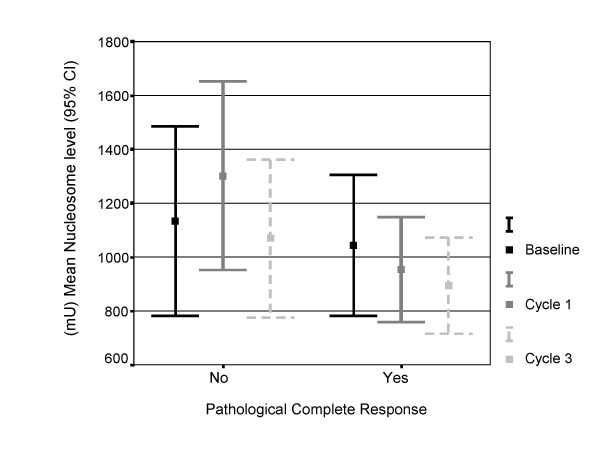
Nucleosome value distribution according to response (expressed as mean and CI 95%). A clear trend for decrease in both measurements is shown in responding patients.

**Figure 3:**
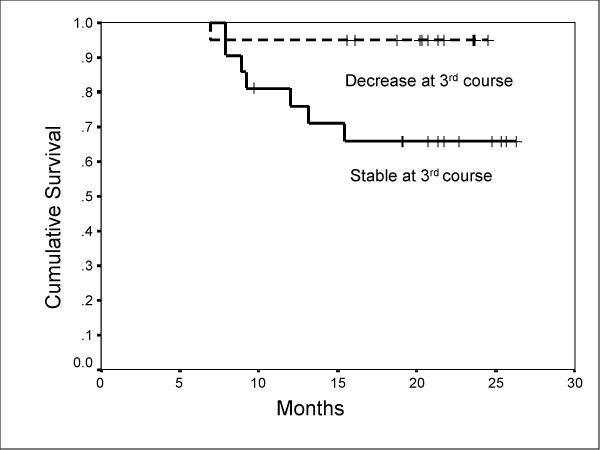
Kaplan-Meier estimates of survival at a mean follow-up of 23 months (range, 7–26 months) according to nucleosome values determined in the third chemotherapy course (*p *= 0.0260). Time to progression-free survival interval was also statistically significant longer in patients who experienced decreased nucleosomes.

On the other hand, Tables 2 and 3 show that nucleosomes at the third – but not at the first – course correlated with pathologic response. This is illustrated in Figure 2, which shows a trend for lower levels of nucleosomes in both first and third courses in patients who achieved complete response with regard to those who did not, suggesting that these tumors were sensitive to chemotherapy and that they failed to develop resistance during subsequent therapeutic courses.

Our results to date demonstrate that decrease in serum nucleosomes during chemotherapy treatment – although marginally – correlates with response under these experimental conditions. To investigate whether the kinetics in nucleosome levels possesses prognostic significance, we analyzed time to progression-free survival and overall survival. Results showed that while nucleosome levels at the first cycle failed to correlate with time to progression and survival, third-cycle levels did correlate with both parameters; on the other hand, earlier stages (IB2-IIB) and smaller tumors also showed longer time to progression and survival. Interestingly, neither time to progression nor survival were correlated with pathologic response. To investigate which variables maintained their prognostic significance for survival, a Cox proportional hazard model was undertaken using pairs of covariates to ascertain which of these pairs better predicted outcome. Nucleosomes determined at the third course and tumor size emerged as statistically significant; however, these results should be considered with caution, as confidence intervals were very wide, perhaps as a result of small sample size and small number of events.

The biological meaning of circulating nucleosomes and the factors that govern their production and clearance from the body are yet to be understood. A body of experimental evidence demonstrates that in mice, apoptosis develops rapidly, within hours, after cytotoxic treatments and that it is dose-dependent [31]; moreover, the extent of apoptosis is higher with the first treatment course than after subsequent doses [30]. In this study, we found that nucleosomes after the first course lowered to a lesser degree than after the third cycle. This finding may reflect either that a single measurement after the initial cycle is not sufficient and would require repeated measurements during the first course of chemotherapy, as recently reported by Holdenrider et al. These investigators found that the area under the curve of nucleosomes during days 1–8 of the first chemotherapy cycle carried the strongest prognostic impact in patients with lung cancer [32].

Our finding on the correlation between nucleosome levels at the third course with prognosis may suggest that the kinetics of circulating nucleosomes may not only reflect the immediate, chemotherapy-induced debulking effect, but that other factors such as amount and/or activity of serum nucleases [33], efficiency of nucleosome clearing from circulation [34,35], and perhaps the macrophage number and function [36] may also influence the behavior of nucleosomes, which in turn may have possess on their own an effect on tumor-host interactions that influences the patient's prognosis. This is suggested by data demonstrating that chromatin fragments impair the natural killer-mediated cytotoxicity of tumor cells [37].

## Conclusion

Serum nucleosome level as determined in the third course of neoadjuvant chemotherapy in patients with cervical cancer was marginally related with tumor response and survival. Our findings, along with those reported by other authors on diverse tumor types, suggest that circulating nucleosome measurements could be a useful predictive and/or prognostic factor in the management of patients with cancer. Further studies are needed to better define not only the predictive/prognostic role of circulating nucleosomes in cancer, but also their potential participation in their own right in response to therapy.

## Competing interests

The author(s) declare that they have no competing interests.

## Authors' contributions

C T-B, L T-C, and A V-C performed sampling and analysis of nucleosomes, while LF O-O carried out the statistical analysis, LC cared for patients, and A D-G conceived the study and wrote the manuscript. All authors read and approved the final manuscript

## Pre-publication history

The pre-publication history for this paper can be accessed here:



## Figures and Tables

**Table 1:** Patient characteristics

Number	41
Age (years)	45 (24–67)
Clinical stage	
IB2-IIB	34 (84)
IIIB	7 (16)
Tumor size (cm^2^)	
<30	22 (53)
>30	19 (47)
Histology	
Squamous	33 (81)
Adeno/adesq	8 (19)

**Table 2:** Correlations of nucleosome levels in the first course and response to chemotherapy

	Complete response	Partial response	p value
Nucleosome decrease	10	10	
Nucleosome increase	6	15	*p *= 0.160

**Table 3:** Correlations of nucleosome levels in the third course and response to chemotherapy

	Complete response	Partial response	p value
Nucleosome decrease	11	9	
Nucleosome increase	5	16	*p *= 0.041

**Table 4:** Cox model for survival

Variable	SE	RR	CI 95%	p value
Nucleosome in third course Decrease vs Stable	1.07	6.86	0.84–56	0.072
Tumor size <30 cm^2 ^vs >30 cm^2^	1.07	9.08	1.10–74	0.040

SE, standard error; RR, response rate. CI 95%, 95% confidence interval.
